# Supplementary figures and images for: Allyl-, Butyl- and Phenylethyl-Isothiocyanate Modulate Akt–mTOR and Cyclin–CDK Signaling in Gemcitabine- and Cisplatin-Resistant Bladder Cancer Cell Lines
Source: Int J Mol Sci. 2022 Sep 20;23(19):10996. doi: 10.3390/ijms231910996 (PMC9570347; doi:10.3390/ijms231910996)

# RT112 - AITC

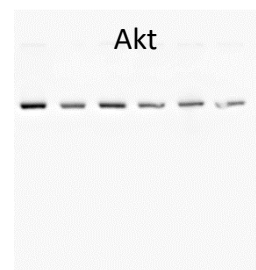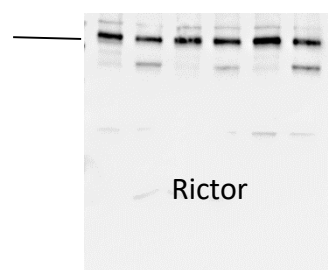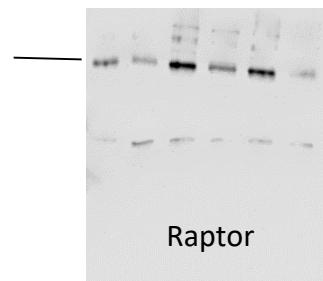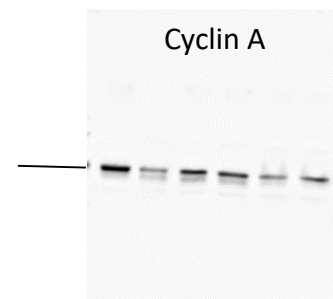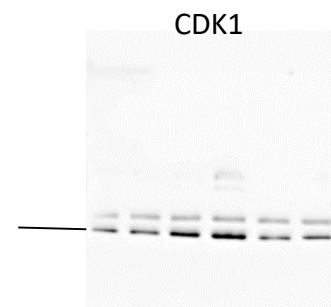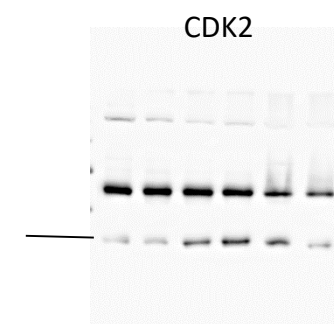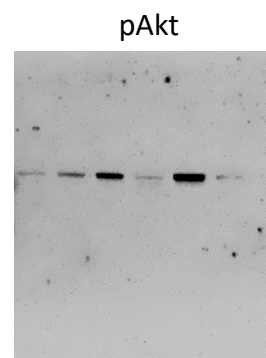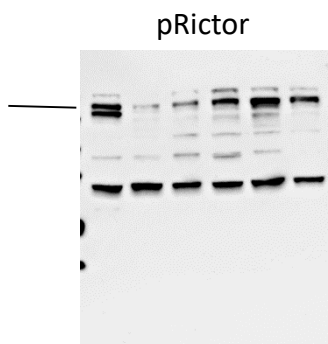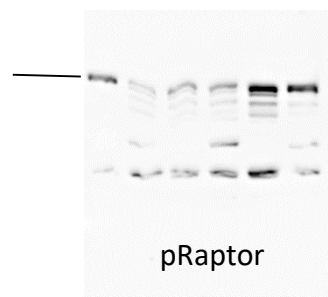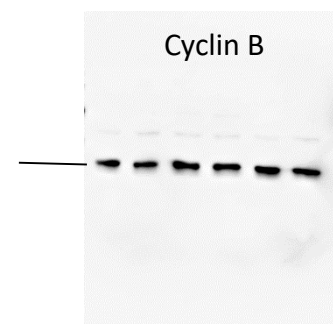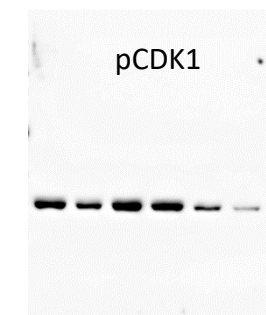

## RT112 – BITC, PEITC (Each: Control, BITC, PEITC)

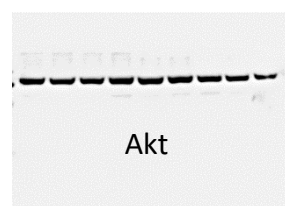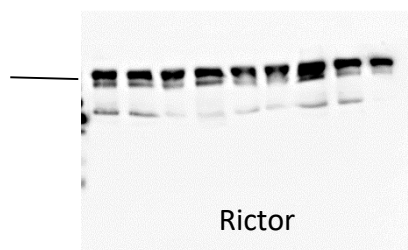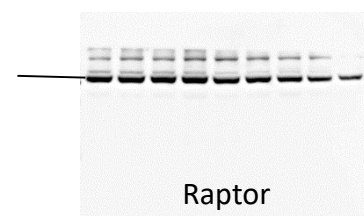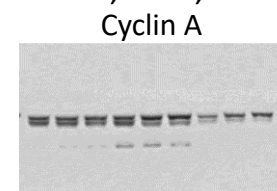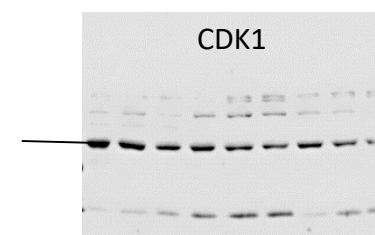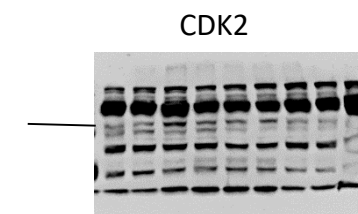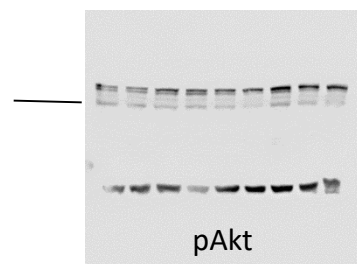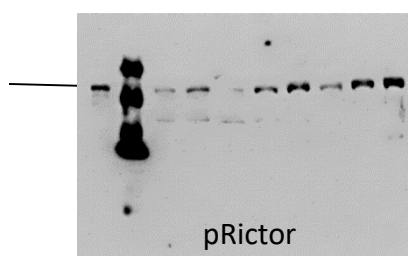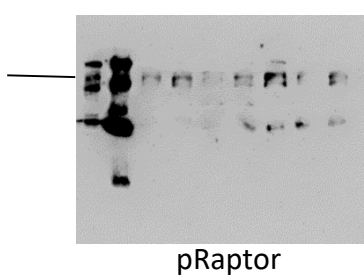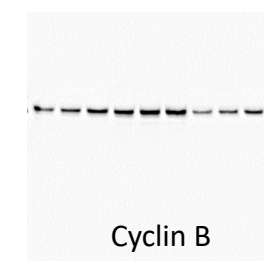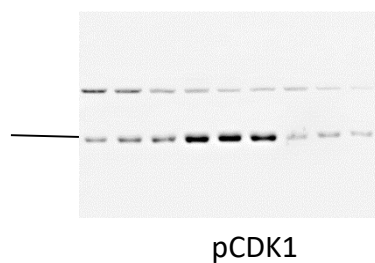

T24 - PEITC

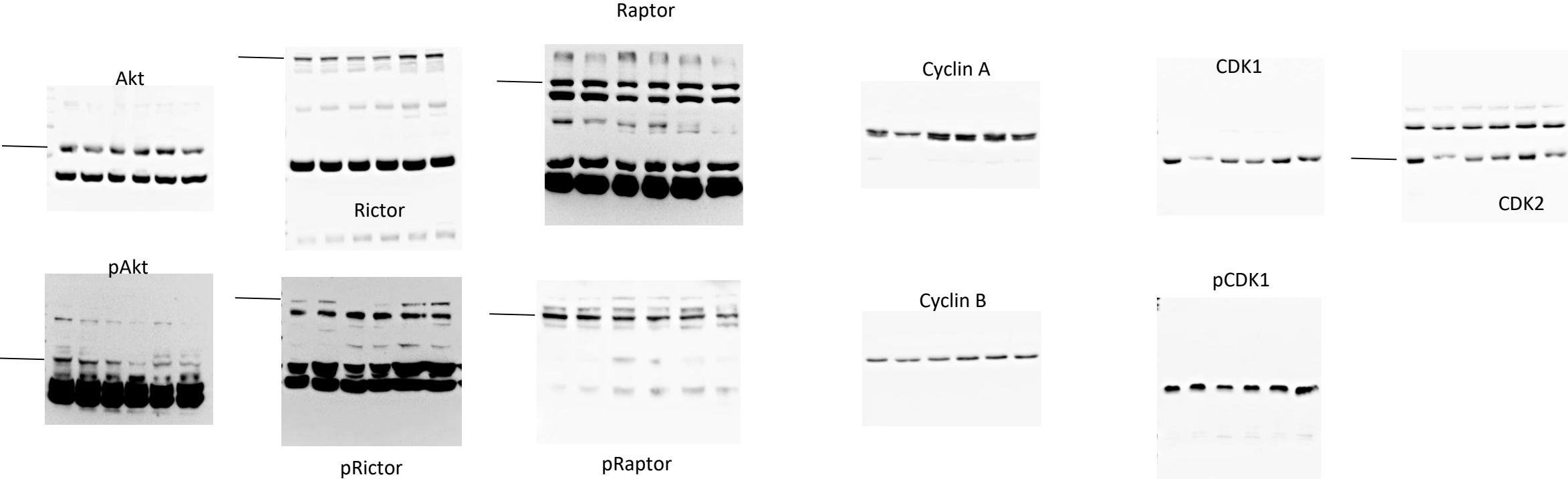

# T24 - BITC

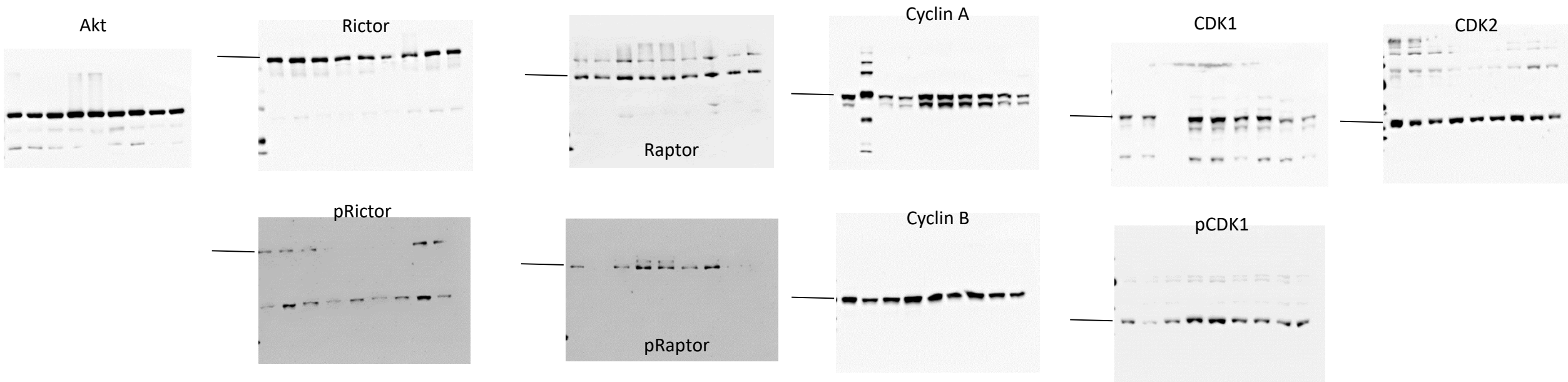

# T24 - AITC

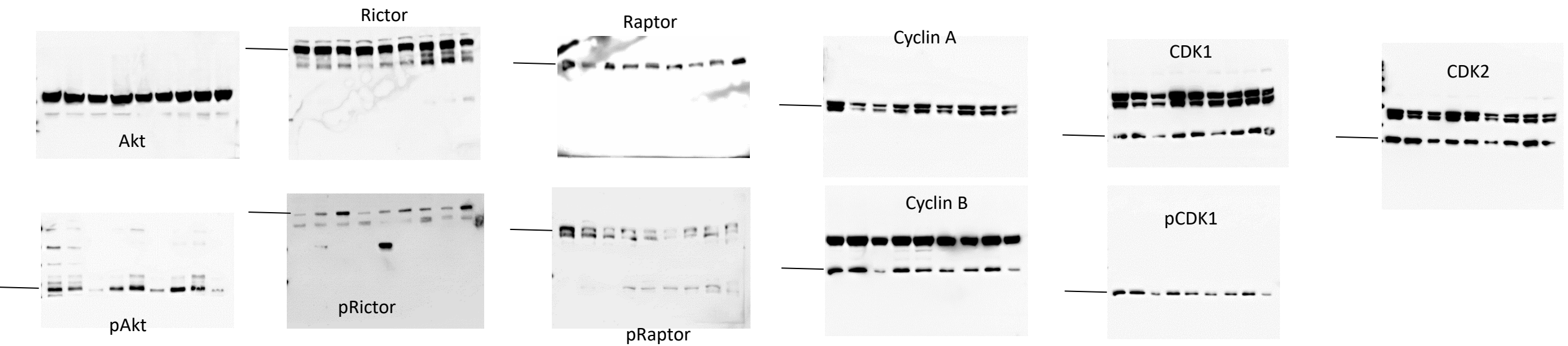

Figure S1: Western blots

Supplement: Supplementary file 1 [file ijms-23-10996-s001.zip › ijms-1899035-supplementary.pdf]
